# Systematic review and meta-analysis of female lifestyle factors and risk of recurrent pregnancy loss

**DOI:** 10.1038/s41598-021-86445-2

**Published:** 2021-03-29

**Authors:** Ka Ying Bonnie Ng, George Cherian, Alexandra J. Kermack, Sarah Bailey, Nick Macklon, Sesh K. Sunkara, Ying Cheong

**Affiliations:** 1 School of Human Development and Health, Faculty of Medicine, University of Southampton, Southampton, SO16 6YD UK; 2 Department of Obstetrics and Gynaecology, Princess Anne Hospital, Room F86, Level F, Coxford Road, Southampton, SO16 5YA UK; 3 Zealand University Hospital, University of Copenhagen, Copenhagen, Denmark; 4 London Women’s Clinic, London, W1G 6AP UK; 5 Department of Women and Children’s Health, Kings College London, Guy’s Hospital, 11th Floor, Tower Wing, London, SE1 9RT UK; 6 Complete Fertility Southampton, Princess Anne Hospital, Coxford Road, Southampton, SO16 5YA UK

**Keywords:** Reproductive disorders, Lifestyle modification, Risk factors

## Abstract

It is known that lifestyle factors affect sporadic miscarriage, but the extent of this on RPL (recurrent pregnancy loss) is less well known. A systematic review and meta-analysis was performed to assess the associations between lifestyle factors and RPL. Studies that analysed RPL in the context of BMI, smoking, alcohol and caffeine intake were included. The primary and secondary outcomes were odds of having RPL in the general population and odds of further miscarriage, respectively. Underweight and women with BMI > 25 are at higher odds of RPL in the general population (OR 1.2, 95% CI 1.12–1.28 and OR 1.21, 95% CI 1.06–1.38, respectively). In women with RPL, having BMI > 30 and BMI > 25 has increased odds of further miscarriages (OR 1.77, 95% CI 1.25–2.50 and OR 1.35, 95% CI 1.07–1.72, respectively). The quality of the evidence for our findings was low or very low. Being underweight and BMI > 25 contributes significantly to increased risk of RPL (general population). BMI > 25 or BMI > 30 increases the risk of further miscarriages (RPL population). Larger studies addressing the effects of alcohol, cigarette smoking and caffeine on the risk of RPL with optimisation of BMI in this cohort of women are now needed.

## Introduction

Spontaneous early pregnancy loss (or miscarriage) is described as any pregnancy that fails to progress beyond 24 weeks, resulting in death and often expulsion of the embryo or fetus^1^. It is the most common complication of early pregnancy, affecting 15–20% of all pregnancies^2^. Recurrent pregnancy loss (RPL) is defined by the European Society of Human Reproduction and Embryology (ESHRE) as 2 or more consecutive miscarriages, occurring in 1–2% of couples^3^. However, many other countries have adopted the term ‘recurrent miscarriage’ (RM), defined as the occurrence of 3 or more consecutive miscarriages occurring in 1% of couples.

RPL is a complex disease where causation has been attributed to numerous factors including those related to chromosomal abnormalities, immunological and immunogenic, endocrinological, DNA fragmentation in the sperm, impairment in the biosensor function of the endometrium as well as lifestyle influences^4^. Standard investigations will be normal for many couples and the cause of RPL is deemed ‘unexplained’ in around 50% of cases.

Lifestyle factors are modifiable and in many instances optimisation of these enhances the chances of a positive reproductive outcome. Whilst the specific mechanisms leading to early pregnancy loss is still relatively unknown, poor lifestyle is associated with a hostile reproductive environment whereby optimal embryo implantation and securement of a pregnancy is compromised^5^. It is now clear that the peri-implantation intrauterine environment is a key determinant of pre-implantation embryo development and early programming^6^. For example, differences in a women’s diet can significantly alter the amino acid milieu within human uterine fluid^7^. The literature studying the effects of various lifestyle factors on RPL has not been comprehensively reviewed and current recommendations^3^ are based on evidence from studies on a population who have had sporadic miscarriages. These findings may not be extrapolated to those with RPL. Isolated miscarriages are associated with an abnormal embryonic karyotype, however as the number of consecutive miscarriage increases, the frequency of abnormal embryonic karyotype significantly reduces^8^. This suggests that the impact of lifestyle may be more significant on the RPL population compared to those with an isolated early miscarriage.

This systematic review and meta-analysis will investigate the impact of female lifestyle factors, namely BMI, smoking, caffeine and alcohol on RPL in the general population as well as further miscarriage in the RPL population. This would help in understanding probable associations to improve patient management.

## Methods

### Sources

Electronic databases: Medline, Embase, Cochrane Library and CINHL were used to conduct a comprehensive search of original and review articles addressing lifestyle and miscarriage history (see Supplementary Method—Search Strategy). The search was limited to studies written in English only. The search included all studies published until March 2020. The lifestyle search terms included ‘diet’, ‘smoking’, ‘alcohol intake’, ‘caffeine intake’, ‘recreational drugs’, ‘exercise’, ‘physical activity’, ‘BMI’, ‘stress’ (physical and mental) and ‘shift work’. The search term used for miscarriage history included ‘early pregnancy loss’, ‘miscarriage’, ‘recurrent pregnancy loss’, ‘recurrent miscarriage’ and ‘spontaneous abortion’. We also used special features and truncations to identify synonyms and broaden the search. We contacted the authors of the studies where necessary for clarification of reported findings. There was no formal attempt to retrieve any unpublished data. References were hand-searched to identify additional references.

### Eligibility

Full text manuscripts were reviewed for relevancy (KYBN and GC). Studies were included if they explored women of reproductive age who had been exposed to an aspect of female lifestyle, such as obesity, being overweight or underweight, smoking, alcohol intake and caffeine intake. We excluded studies which assessed the effectiveness of an intervention aimed at altering lifestyle and studies which assessed lifestyle factors in the context of IVF outcomes or associations with PCOS. The primary outcome assessed was the risk of having RPL in the general population. The secondary outcome was the risk of having a further miscarriage in the RPL population. We define RPL as 2 or more consecutive spontaneous early pregnancy losses (or miscarriages). We define spontaneous early pregnancy loss as any pregnancy that fails to progress beyond 24 weeks.

We screened all studies, reviewing full papers where required and disregarding those that did not meet the eligibility criteria using a double-blind approach. All studies were assessed by KYBN and GC independently and any disagreement was discussed and then a decision made for inclusion or exclusion. A third reviewer was called to settle any disagreements where necessary. Authorships and data sources were crosschecked and any duplications were excluded.

### Quality of included studies

Each study was assessed for quality of parameters including study design, population size, RPL definition, classification of lifestyle parameter, and miscarriage confounding factors. These are detailed in the Supplementary Table S1. We also used the grading system developed by the Grading of Recommendations, Assessment, Development and Evaluation (GRADE) group to assess the quality of the evidence for the outcome of RPL risk or risk of further miscarriage in relation to female lifestyle exposures (Table 1)^9^.

**Table 1:** GRADE analysis of the evidence used in the meta-analyses.

Outcomes	Relative effect (95% CI)	No of participants (studies)	Quality of evidence (GRADE)
Further miscarriage in RPL population in BMI > 30	OR 1.77 (1.25–2.50)	803 (2 studies)^19,21^	Low^a^
Further miscarriage in RPL population in BMI > 25	OR 1.35 (1.07–1.72)	1101 (2 studies)^19,21^	Low^a^
RPL in general population in BMI > 25	OR 1.21 (1.06–1.38)	67,911 (2 studies)^14,15^	Low^a^
Further miscarriage in RPL population in underweight BMI	OR 0.65 (0.04–11.65)	651 (2 studies)^19,21^	Very low^a,b,c^
RPL in general population in underweight BMI	OR 1.2 (CI 1.12–1.28)	78,661 (3 studies)^14,15,22^	Very low^a,b^
RPL in general population in cigarette smokers	OR 1.62 (0.90–2.93)	1670 (3 studies)^14,22,27^	Very low^a,b^
RPL in general population with alcohol intake	OR 1.12 (0.88–1.44)	1685 (3 studies)^14,23,27^	Low^a^
RPL in general population with caffeine intake	OR 1.35 (0.83–2.19)	1417 (2 studies)^14,27^	Low^a^

^a^All observational studies.

^b^Wide variation in the effect estimates across studies.

^c^Number of events too low in study group to detect precise estimate of effect.

### Data analysis

The association of female lifestyle factors and risk of RPL are presented in this review. Results from all studies that fulfilled the inclusion criteria were summarised together with the information about the study (Supplementary Tables S1–S4). Authors were contacted for extra data where possible.

Results from different study designs were reviewed separately. Individual study estimates were pooled using random effects (if heterogeneity, I^2^ ≥ 50%) or fixed effects (if heterogeneity, I^2^ < 50%) meta-analysis^10^. Mantel–Haenszel was the statistical test used for the meta-analyses as it allows for the investigation of association between a binary predictor and binary outcome for observational studies^10^. Results are presented as odds ratios (ORs) and 95% confidence intervals (95% CI) for the association between lifestyle factors and risk of RPL in the general population or risk of further miscarriage in the RPL population. Results were deemed as significant when P < 0.05. For statistical analysis and generation of forest plots, RevMan version 5.4 (Copenhagen: The Nordic Cochrane Centre, The Cochrane Collaboration, 2014) was used. Where the data were recorded in differing methods or units, a meta-analysis was not performed. In these cases a narrative summary of the findings was carried out.

Where there were different definitions of RPL, datasets were combined together for meta-analyses if the study included participants who had experienced two or more miscarriages.

For meta-analysis of the association between BMI and RPL a subgroup analysis was performed. Datasets were combined for BMI > 30 and separately for datasets of BMI > 25. Papers differed in their definition of ‘underweight’ and therefore the lower end of ‘normal’ BMI (18.5, 19 and 20), resulting in a discrepancy of up to a BMI of 1.5. Despite this, the ‘underweight’ and ‘normal’ BMI categories from different papers were combined for the purposes of meta-analyses. With the effect of BMI on RPL, a distinction was made in the analysis of RPL within the general population and further miscarriage in the RPL population.

Analysis of the effect of caffeine intake, cigarette smoking and alcohol consumption on RPL looked at RPL within the general population. For meta-analysis of the association between caffeine intake and RPL, datasets were combined for lower caffeine intake of < 99 mg/day and higher caffeine intake of > 99 mg/day. For cigarette smoking, datasets of non-smokers and current smokers were combined. For consumption of alcohol, datasets of non-drinkers and women who consumed alcohol were combined.

## Results

### Search results

**Figure 1:**
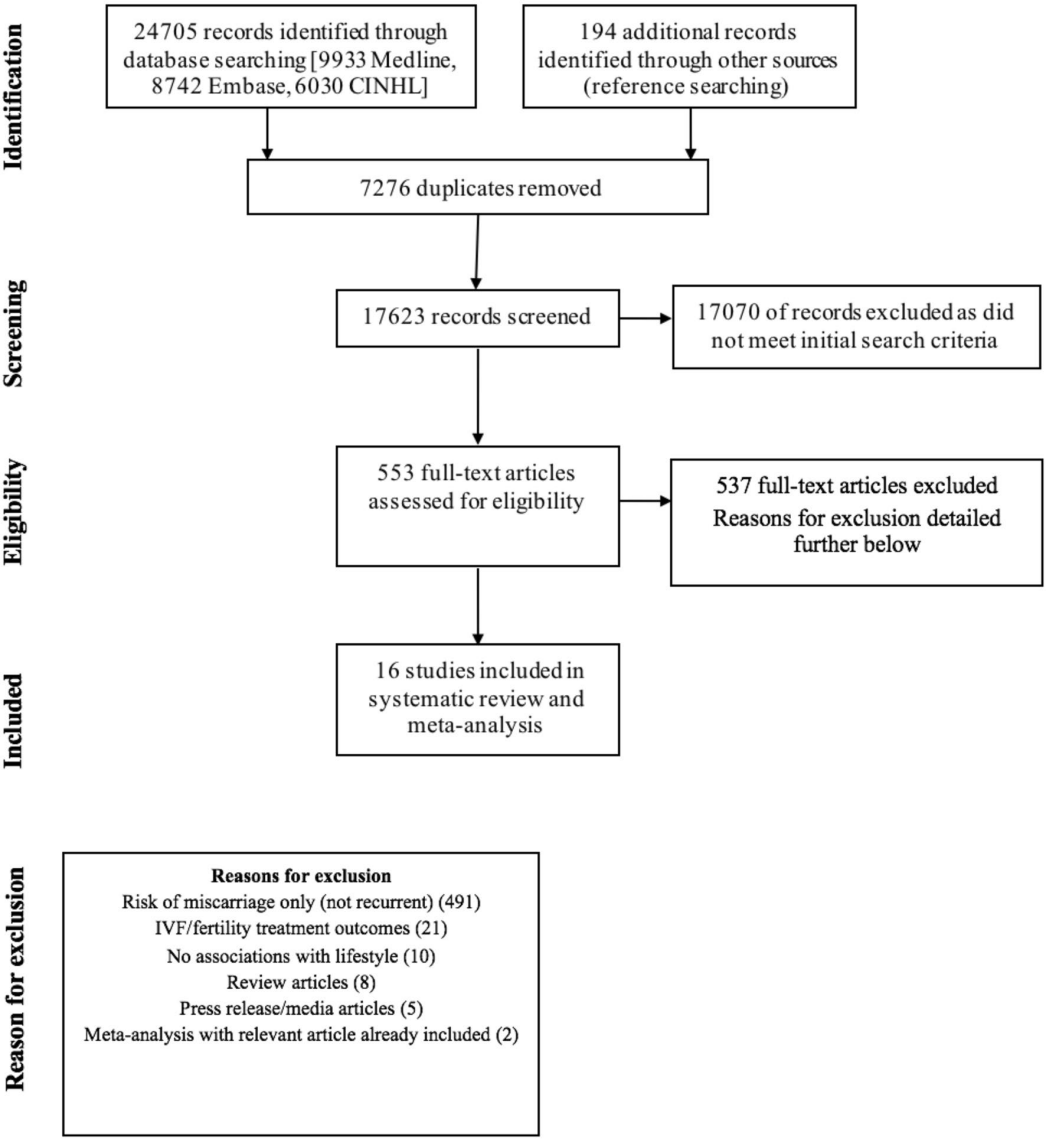
PRISMA flow diagram detailing search results^11^.

The PRISMA flow diagram details our search results (Fig. 1)^11^. The systematic search identified a total of 24, 705 records and an additional 194 records through hand-searching of references. 16 studies were included in this systematic review and meta-analysis^12–27^. None of these studies were RCTs; there were 8 case control studies, 6 cohort studies, 1 survey-based study and 1 cross-sectional study. Supplementary Tables S1 to S4 summarise the studies that have been included and the participant characteristics. The quality of the evidence was low or very low, mainly due to the inconsistencies of results from a small number of studies and heterogeneity in study populations. Some studies investigated the general population and the risk of RPL based on different lifestyle factors. Others recruited only women who were known to have RPL and studied the effect of lifestyle factors on further miscarriage. Therefore, the analysis was grouped separately for the general population and the RPL population.

### Overweight and obesity

Supplementary Table S2a summarises the ten studies that have addressed RPL and high BMI and the BMI cut-offs used for each one^12–15,17,19–21,25,27^.

**Figure 2:**
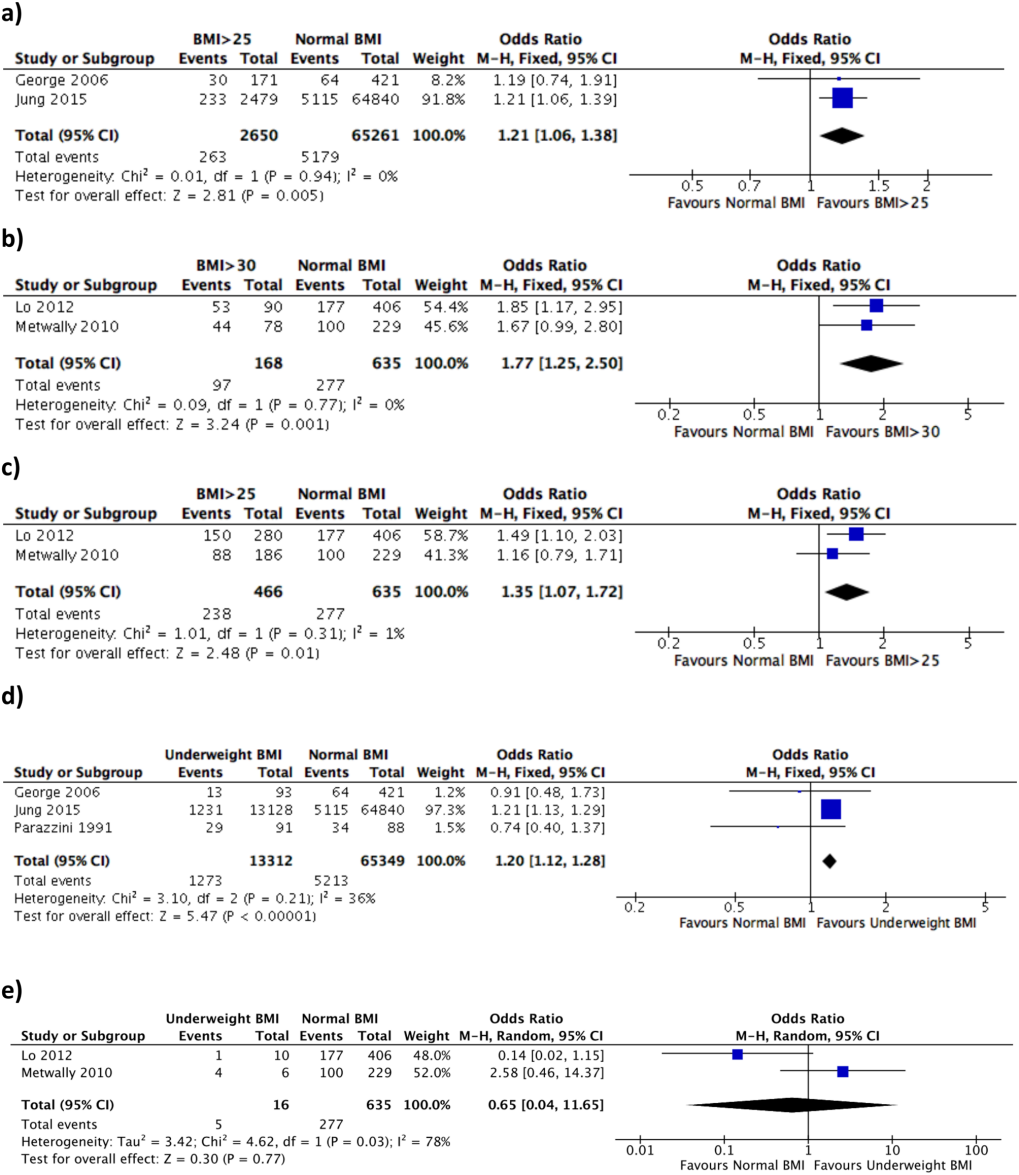
Forest plot demonstrating meta-analysis of the effect of BMI on RPL and further miscarriage. (**a**) BMI > 25 significantly increases risk of RPL in general population. (**b**) BMI > 30 significantly increases risk of further miscarriage in RPL population. (**c**) BMI > 25 significantly increases risk of further miscarriage in RPL population. (**d**) Underweight BMI significantly increases risk of RPL in general population. (**e**) Underweight BMI has no effect on risk of further miscarriage in RPL population. 95% CI = 95% confidence interval; M-H = Mantel–Haenszel statistical test.

Comparable studies were combined for meta-analyses. Meta-analysis of 2 studies^14,15^ showed that within the general population, the odds of having RPL is significantly higher in women with BMI > 25 compared to those with normal BMI (OR 1.21, 95% CI 1.06–1.38, P = 0.005) (Fig. 2a). In studies within the RPL population^19,21^, the odds of having a further miscarriage is significantly higher in the BMI > 30 and BMI > 25 sub-groups compared with normal BMI (OR 1.77, 95% CI 1.25–2.50, P = 0.001 and OR 1.35, 95% CI 1.07–1.72, P = 0.01, respectively) (Fig. 2b,c). All the studies used in this meta-analysis were observational; therefore, the quality of evidence of the association between high BMI and RPL in the general population and further miscarriage in the RPL population is low (Table 1).

George et al. and Stefanidou et al. reported no significant difference in risk of RPL with increased BMI in the general population^14,25^. However, four other studies demonstrated a significant association between raised BMI and risk of RPL^15,17,20,27^. RPL was found to be more common if women were obese at the ages of 18–20^15^. The risk of further miscarriage in women with RPL was found to be significantly higher in the obese but not overweight group^13,19,21^. Asian women with a similar BMI to Caucasian women had a significantly increased risk of further miscarriage^19^. Bhandari et al. demonstrated that obese women with RPL were more likely to conceive quicker^12^. PCOS, impaired glucose tolerance and type 2 diabetes are likely surrogates for elevated BMI, which is seen in over 70% of women with RPL^20^.

### Underweight

Supplementary Table S2b summarises the six studies that have addressed associations between RPL and low BMI and the BMI cut offs used for each one^14,15,19,21,22,26^.

Figure 2d,e show meta-analyses of two different groups of studies. Being underweight compared to normal BMI significantly increases the odds of RPL within the general population (OR 1.2, 95% CI 1.12–1.28, P < 0.00001) but not the odds of further miscarriage within the RPL population and OR 0.65, 95% CI 0.04–11.65)^14,15,19,21,22^. The quality of the evidence for the association of being underweight and the risk of RPL in the general population and the risk of further miscarriage in the RPL population was very low (Table 1). We downgraded a further evidence level from low based on unexplained variability in results across the small number of included studies and low number of events in the study group.

George et al. and Parazzini et al. found no association between risk of RPL and being underweight in the general population^14,22^. However, Ticconi et al. reported that RPL was associated with a mean lower BMI and Jung et al. found that women who were underweight between the ages of 18–20 were more likely to experience RPL compared to women with a normal BMI^15,26^. Metwally et al. reported that risk of further miscarriage within the RPL population was significantly higher in underweight women but in contrast to this Lo et al. found that underweight women with RPL were no more likely to be affected by a further miscarriage^19,21^.

### Smoking

Studies looking at the effect of tobacco smoking on risk of RPL in the general population have been inconsistent; Supplementary Table S3a summarises the five studies^14,22,24,25,27^.

**Figure 3:**
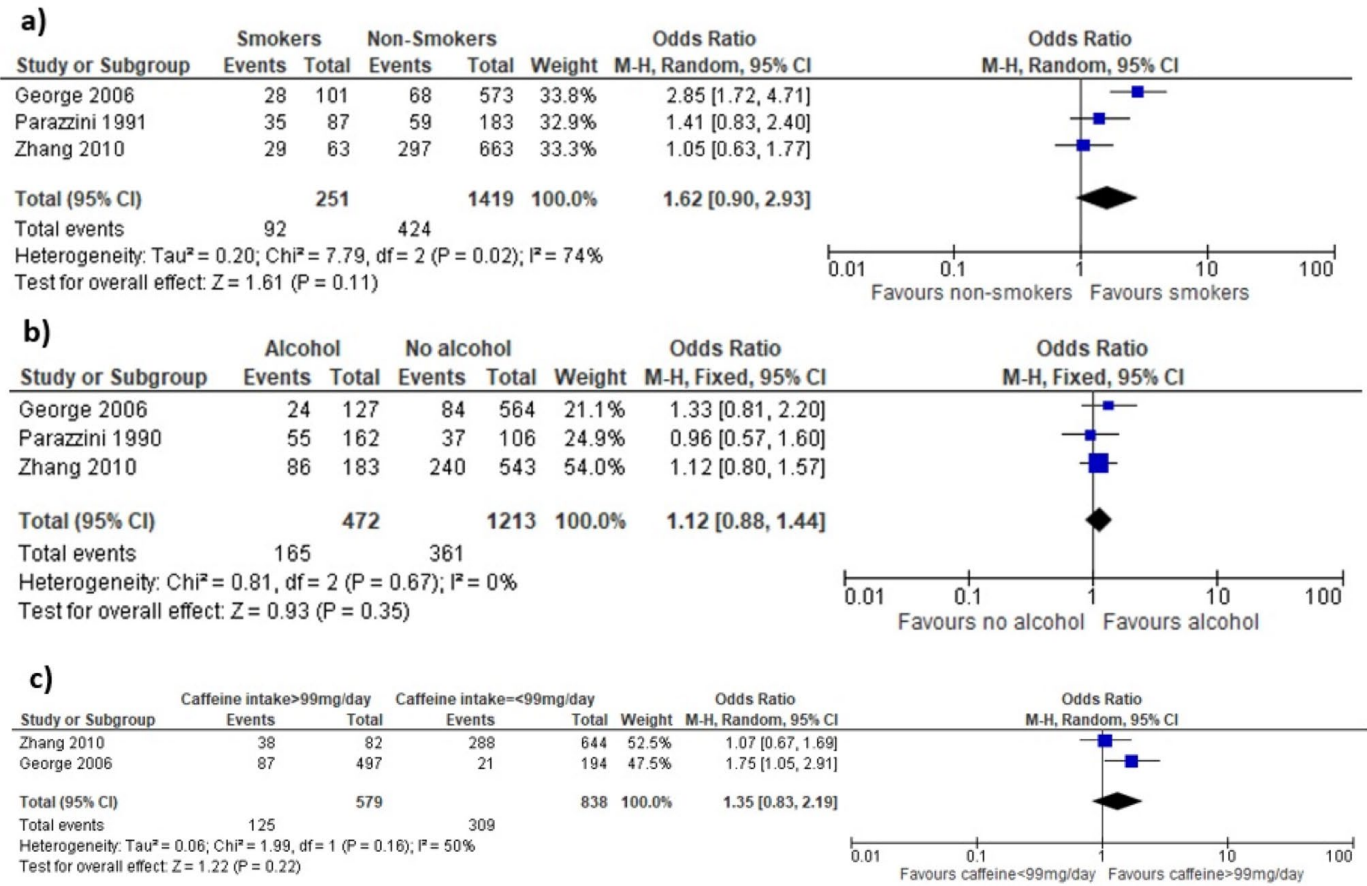
Forest plot demonstrating meta-analysis of the effect of lifestyle factors on RPL. (**a**) Cigarette smoking does not significantly increase risk of RPL in general population. (**b**) Alcohol intake does not significantly increase risk of RPL in general population. (**c**) Caffeine intake does not significantly increase risk of RPL in general population. 95% CI = 95% confidence interval; M-H = Mantel–Haenszel statistical test.

Figure 3a shows meta-analyses of three studies. The odds of RPL in the general population is increased in women who were cigarette smokers compared to non-smokers but this did not reach statistical significance (OR 1.62, 95% CI 0.90–2.93)^14,22,27^. Stefanidou et al. reported significant association between smoking and RPL risk; however, we have not included this study in the meta-analysis as the raw data does not correspond with the OR that has been reported and we were not able to contact the authors for clarification of the data^25^. The quality of evidence for the association between smoking and RPL risk was very low because of inconsistency of results across the small number of studies included (Table 1). Due to heterogeneity in the quantification and reporting of smoking behaviours, we were only able to include three studies in our meta-analysis.

Three of the studies found that cigarette smoking significantly increased the risk of RPL within the general population^14,25^. Other studies have shown that the effects of smoking in elevating the risk of RPL were non-significant, although the effects increased with the amount of cigarettes smoked per day and this trend was significant^22,27^. One study has also shown a significant association between RPL and exposure to second-hand cigarette smoke^24^. There were no studies assessing the association between RPL risk and vaping.

### Alcohol

Supplementary Table S3b summarises the findings from four studies assessing associations in alcohol intake and RPL in the general population^14,23,25,27^. Consumption of all types of alcohol were included and was defined as the number of drinks or units consumed on average depending on the study.

Figure 3b shows meta-analyses of three studies. Alcohol intake compared with no alcohol intake increases the risk of RPL, however this is not statistically significant (OR 1.12, 95% CI 0.88–1.44)^14,22,27^. The quality of the evidence for the association between alcohol intake and RPL risk was low as they were all observational studies (Table 1). Different methods for reporting the quantity of alcohol intake amongst the studies precluded further detailed analyses of effects of different alcohol intake limits.

All four studies demonstrated that alcohol does not have a statistically significant effect on the risk of RPL within the general population^14,23,25,27^. Stefanidou et al. reported that women with RPL consumed less alcohol than controls and the difference was significant; however, the odds ratio reported does not correspond with the raw data provided and we were not able to contact the authors for clarification^25^. We therefore interpret this with caution and have not included it in our meta-analyses.

### Caffeine

Supplementary Table S3c summarises the four studies that have addressed associations between RPL and caffeine intake^14,23,25,27^.

Figure 3c shows meta-analyses of 2 studies comparing risk of RPL in women who have higher caffeine intake (> 99 mg/day) and women who have lower caffeine intake (≤ 99 mg/day)^14,27^. The odds of RPL in the higher caffeine intake group compared to the lower caffeine intake group is higher but not significant (OR 1.35, 95% CI 0.83–2.19). The quality of evidence for the association between caffeine intake and RPL risk was low as they were all observational studies (Table 1). Different methods for reporting the quantity of caffeine intake amongst the studies precluded further detailed analyses of effects of different caffeine intake limits. Studies by Parazzini et al. and Stefanidou et al. were not included as data for caffeine intake was not presented in a way that could be combined for meta-analyses^23,25^.

Caffeine has been shown to increase the risk of RPL in a dose-dependent manner, with consumption of > 300 mg caffeine/day being associated with the highest risk^14,25^. The study by George et al. reported that the effect of caffeine was not demonstrated in smokers^14^. Other studies have shown that caffeine consumption has no effect on the risk of RPL within the general population^23,27^.

### Stress

Supplementary Table S4a summarises the findings on stress and RPL. Women with RPL had higher and more variable scores on questionnaires designed to measure perceived stress^18^. Among women with RPL, the ones who had a subsequent live birth had increased depression compared to those who had another miscarriage, and the authors suggest that moderate stress may be beneficial to future pregnancy outcome. Kolte et al. found a significantly increased odds of having moderate or severe depression (OR 5.53, 95% CI 2.09, 14.61) and high stress levels (OR 1.59, 95% CI 1.03, 2.44) in women with RPL compared to those who did not^16^. However, with both studies it is not possible to determine whether increased stress is the result of RPL or a contributing factor to RPL (i.e. stress being an exposure prior to pregnancy losses).

### Nutrition supplement

Only one study (Supplementary Table S4b) assessed association between nutritional supplements and RPL^14^. Although plasma folate levels were not significantly associated with repeat miscarriages, women taking folate supplements were found to have an increased risk of RPL compared to women who were not taking daily folic acid supplements (OR 3.1, 95% CI 1.4, 6.6) in crude analyses. However, in this cohort, women taking folic acid supplements were significantly older and took longer to conceive compared to non-supplement takers.

### Shift work

George et al. found no increased risk of RPL in shift workers (OR 1.3, 95% CI 0.5–3.0) (Supplementary Table S4c)^14^.

## Discussion

In this systematic review and meta-analysis, we showed that the odds of having RPL in the general population is increased in the underweight and BMI > 25 subgroups by 1.2-fold compared to those with normal BMI. The risk of having further miscarriage in the RPL population is higher for raised BMI and this effect is further exaggerated when performing subgroup analyses on women with a BMI of > 30; there is a 1.3-fold and 1.7-fold increase in risk of further miscarriage in the BMI > 25 and BMI > 30 subgroups respectively. Meta-analyses of other lifestyle risk factors, including alcohol intake, cigarette smoking and higher caffeine intake have shown no increased risk of RPL, although evidence is limited to very few studies. All studies in this review were observational, with the quality of the evidence presented being low or very low^9^. This implies that the true effect may differ from the estimate (when evidence is low) or the true effect is likely to be substantially different from the estimated effect (when evidence is very low). The main limiting factor in the quality of the evidence is clinical heterogeneity and inconsistency of results across a small number of studies. We did not identify any RCTs from this review.

Optimising lifestyle parameters in the peri-conceptional period has been the focus of management of reproductive failures including RPL and infertility^3,28^. The epigenetic effects on the fetus and the transgenerational effects has provided even stronger arguments for optimising lifestyle in early pregnancy establishment. Studies have shown significant impacts of preconception maternal diet on fecundity; lower intakes of fruit and higher intakes of fast food are both associated with longer time to pregnancy and infertility^29^. Although there have been meta-analyses on lifestyle and its effects on fertility and sporadic miscarriage or spontaneous abortion, the effect of modifiable factors, including lifestyle on RPL has been less well explored. The cause of RPL remains elusive, and although commonly clinicians and patients look to optimising lifestyle, there is limited data available on lifestyle impacts in the cohort of patients affected by RPL.

In this review we have identified BMI > 25 to be a significant modifiable risk factor for RPL within the general population and further miscarriage within the RPL population, compared to those with normal BMI. These results are in agreement with an earlier meta-analysis performed which included 2 studies, showing an increased odds of further miscarriage in the RPL group in obese women compared to those with normal BMI (OR 1.75, 95% CI 1.24–1.70)^30^. The effects of being overweight and obese are exaggerated in the RPL population compared to the population with an isolated miscarriage; a meta-analysis of 32 studies identified an increase in the relative risk (RR) of clinical miscarriage in overweight women (RR 1.09, 95% CI 1.04–1.13) and obese women (RR 1.21, 95% CI 1.15–1.27)^31^. Obesity has significant impacts on female reproductive health and an increased BMI is associated with infertility, poor outcomes after fertility treatment, and pregnancy loss^32,33^. Although gradual weight loss has been shown to improve fertility outcomes^33^, there have been no studies to date assessing the effect of weight loss on RPL. Moreover, the exact cause of obesity related increase risk of RPL is unknown. An unfavourable endometrial milieu associated with obesity is one possible explanation, and the possibility of oocyte abnormality in obese women is another explanation^34^. However, the latter has been refuted by a study of obese women receiving oocyte donation who experienced a higher rate of miscarriage compared with those of normal BMI^35^. There are also various immunological pathways that may play a role in miscarriage. For example, high levels of C-reactive protein (CRP) and interleukin- 6 (IL-6) are present in obesity as well as women with RPL^36–38^, and this state of ‘chronic inflammation’ may contribute to impaired implantation and placentation, as well as complications in pregnancy and post-partum^39^. It is likely that unfavourable reproductive environmental exposures within obese women negatively impact the development of the oocyte, the ability for oocyte to be fertilised and the ability for healthy embryo implantation and development.

One large case control study has previously shown that being underweight is associated with sporadic first trimester miscarriage^40^. However, when focussing on the risk of RPL the evidence remains conflicting and the meta-analysis performed in this study shows an increase in RPL risk in underweight women in the general population but no increase in the risk of further miscarriage in the RPL population. The quality of evidence is very low given the inconsistency in findings across a small number of studies.

Our review has shown no increased risk of RPL in the general population in cigarette smokers compared with non-smokers, in women who consume alcohol compared to those who do not consume alcohol, or in women with a higher level of caffeine intake compared to lower intake. We were not able to further stratify risk based on quantity of cigarettes smoked, intake of alcohol or caffeine due to variations in recording of data between studies. A systematic review performed showed that cigarette smoking does mildly increase the risk of sporadic miscarriage (OR 1.23, 95% CI 1.16–1.30) and that exposure to secondhand smoke increases the risk of miscarriage by 11%^41^. Several components of tobacco smoke are toxic for the fetus; these include nicotine, carbon monoxide and cyanide. Nicotine reduces blood flow through the placenta through its vasocontrictive effects, and carbon monoxide binds to haemoglobin, causing both fetal and maternal hypoxia, which may interfere with the development of the conceptus^42^. We did not identify any studies that assessed the associations between e-cigarettes/vaping and RPL risk in this review. Further studies exploring the impact of e-cigarettes and vaping on miscarriage and RPL risk are warranted, especially as these behaviours are increasingly common in women and their partners who are attempting to either reduce or stop cigarette smoking with the aim of achieving a successful pregnancy. The data for alcohol consumption has been inconsistent but various studies have suggested that alcohol consumption during pregnancy increases the risk of miscarriage, with an upper limit of 2–4 drinks per week^43,44^. Additionally, a recent systematic review of quasi-experimental studies have shown a likely causal detrimental role of prenatal alcohol exposure on cognitive outcomes and effects on lowered birth weights^45^; hence the latest guidance from the NHS is to abstain from alcohol whilst trying to get pregnant, as well as through the duration of the pregnancy^46^. Caffeine crosses the placenta, reaching the fetus whose metabolic rate is low secondary to low enzyme levels. Previous studies have associated increasing caffeine intake (especially at a level more than 300 mg/day) with early and late miscarriage and stillbirth risk^47–49^. However, in this review we did not perform meta-analyses on caffeine intake of > 300 mg/day as the two studies included had very low numbers of women with this level of caffeine intake.

The main limitations of this systematic review and meta-analyses is the diversity in the methodology and methods of reporting RPL. Although most studies included women with ‘three or more consecutive miscarriages’, some studies included women with ‘two or more miscarriages’ and some have not been strict in whether it is consecutive. The heterogeneity of the populations may also limit conclusions drawn from our study. For example, the confounding effects of diabetes or PCOS have not been addressed and may affect the risk of RPL. The data captured by studies in this review relate to the lifestyle reported at the time of the study and does not capture the pre-pregnancy lifestyle parameters and whether there have been any behavioural modifications. This systematic review was also limited to studies in English. We did not explore the impact of male lifestyle parameters on the risk of RPL.

Despite the limitations, we have shown that several lifestyle parameters are associated with risk of RPL in the general population and further miscarriage in the RPL population. Further large observational or clinical studies are required to delineate the true effects. The biological mechanisms of possible effect also need exploring. For example, the immunological contributions of increased BMI to RPL risk, and the effect of interventions aimed at weight loss in overweight/obese women with history of RPL are areas still needing review.

## Conclusion

Being underweight and having BMI > 25 contributes significantly to RPL in the general population by 1.2-fold. BMI > 25 also significantly increases the risk of further miscarriage in the RPL population by 1.2-fold and in women with BMI > 30 the risk of further miscarriage is increased by over 1.7-fold. Although our systematic review and meta-analysis has not shown an increased risk of RPL with lifestyle parameters such as smoking, alcohol intake and higher caffeine intake, these require further exploration. Current studies are heterogeneous and there remain difficulties in pooling the data due to inconsistencies in methodology and reporting. There is a need for larger observational or clinical studies addressing the dose effects of alcohol, cigarette smoking and caffeine in this cohort of patients. Studies addressing the impact of lifestyle interventions in the RPL population would also be of benefit to improve patient management.

## Supplementary Information

Supplementary Information.

## References

[1] Christiansen OB (2005). Evidence-based investigations and treatments of recurrent pregnancy loss. Fertil. Steril..

[2] Wang X (2003). Reproductive endocrinology: Conception, early pregnancy loss, and time to clinical pregnancy: A population-based prospective study. Fertil. Steril..

[3] ESHRE Early Pregnancy Guideline Development Group. Recurrent Pregnancy Loss. Version 2 (2017).

[4] Larsen EC, Christiansen OB, Kolte AM, Macklon NS (2013). New insights into mechanisms behind miscarriage. BMC Med..

[5] Robertson SA, Chin PY, Femia JG, Brown HM (2018). Embryotoxic cytokines-potential roles in embryo loss and fetal programming. J. Reprod. Immunol..

[6] Barker DJ (1990). The fetal and infant origins of adult disease. BMJ.

[7] Kermack AJ (2015). Amino acid composition of human uterine fluid: Association with age, lifestyle and gynaecological pathology. Hum. Reprod..

[8] Ogasawara M, Aoki K, Okada S, Suzumori K (2000). Embryonic karyotype of abortuses in relation to the number of previous miscarriages. Fertil. Steril..

[9] The Grading of Recommendations, Assessment, Development and Evaluation (GRADE) working group. The GRADE system. https://www.gradeworkinggroup.org (2000).

[10] Deeks, J.J., Higgins, J.P.T. & Altman, D.G. Cochrane Handbook for Systematic Reviews of Intervention Chapter 10: Analysing data and undertaking meta-analyses https://training.cochrane.org/handbook/current/chapter-10.

[11] Moher D, Liberati A, Tetzlaff J, Altman DG, The PRISMA Group (2009). Preferred reporting items for systematic reviews and meta-analyses: The PRISMA statement. PLoS Med..

[12] Bhandari HM, Tan BK, Quenby S (2016). Superfertility is more prevalent in obese women with recurrent early pregnancy miscarriage. BJOG.

[13] Boots CE, Bernardi LA, Stephenson MD (2014). Frequency of euploid miscarriage is increased in obese women with recurrent early pregnancy loss. Fertil. Steril..

[14] George L, Granath F, Johansson AL, Olander B, Cnattingius S (2006). Risks of repeated miscarriage. Paediatr. Perinat. Epidemiol..

[15] Jung SJ (2015). Body mass index at age 18–20 and later risk of spontaneous abortion in the Health Examinees Study (HEXA). BMC Pregnancy Childbirth..

[16] Kolte AM, Olsen LR, Mikkelsen EM, Christiansen OB, Nielsen HS (2015). Depression and emotional stress is highly prevalent among women with recurrent pregnancy loss. Hum. Reprod..

[17] Lashen H, Fear K, Sturdee DW (2004). Obesity is associated with increased risk of first trimester and recurrent miscarriage: Matched case-control study. Hum. Reprod..

[18] Li W, Newell-Price J, Jones GL, Ledger WL, Li TC (2012). Relationship between psychological stress and recurrent miscarriage. Reprod. Biomed. Online..

[19] Lo W (2012). The effect of body mass index on the outcome of pregnancy in women with recurrent miscarriage. J. Fam. Community Med..

[20] Matjila MJ, Hoffman A, van der Spuy ZM (2017). Medical conditions associated with recurrent miscarriage—Is BMI the tip of the iceberg?. Eur. J. Obstet. Gynecol. Reprod. Biol..

[21] Metwally M, Saravelos SH, Ledger WL, Li TC (2010). Body mass index and risk of miscarriage in women with recurrent miscarriage. Fertil. Steril..

[22] Parazzini F (1991). Risk factors for spontaneous abortion. Int. J. Epidemiol..

[23] Parazzini F, Bocciolone L, La Vecchia C, Negri E, Fedele L (1990). Maternal and paternal moderate daily alcohol consumption and unexplained miscarriages. Br. J. Obstet. Gynaecol..

[24] Peppone LJ (2009). Associations between adult and childhood secondhand smoke exposures and fecundity and fetal loss among women who visited a cancer hospital. Tob. Control..

[25] Stefanidou EM, Caramellino L, Patriarca A, Menato G (2011). Maternal caffeine consumption and sine causa recurrent miscarriage. Eur. J. Obstet. Gynecol. Reprod. Biol..

[26] Ticconi, C. *et al.* Body mass index and recurrent pregnancy loss. *Reprod. Sci*. (2010).

[27] Zhang BY (2010). Risk factors for unexplained recurrent spontaneous abortion in a population from southern China. Int. J. Gynaecol. Obstet..

[28] National Institute for Health and Care Excellence (NICE). Fertility problems: Assessment and treatment clinical guideline. http://www.nice.org.uk/guidance/cg156 (2013). 32134604

[29] Grieger JA (2018). Pre-pregnancy fast food and fruit intake is associated with time to pregnancy. Hum. Reprod..

[30] Cavalcante MB, Sarno M, Peixoto AB, Araujo Junior E, Barini R (2019). Obesity and recurrent miscarriage: A systematic review and meta-analysis. J. Obstet. Gynaecol. Res..

[31] Balsells M, Garcia-Patterson A, Corcoy R (2016). Systematic review and meta-analysis on the association of prepregnancy underweight and miscarriage. Eur. J. Obstet. Gynecol. Reprod. Biol..

[32] Metwally M, Ong KJ, Ledger WL, Li TC (2008). Does high body mass index increase the risk of miscarriage after spontaneous and assisted conception? A meta-analysis of the evidence. Fertil. Steril..

[33] Pandey S, Maheshwari A, Bhattacharya S (2010). Should access to fertility treatment be determined by female body mass index?. Hum. Reprod..

[34] Cheong Y, Sadek KH, Bruce KD, Macklon N, Cagampang FR (2014). Diet-induced maternal obesity alters ovarian morphology and gene expression in the adult mouse offspring. Fertil. Steril..

[35] Hegaard HK, Ersboll AS, Damm P (2016). Exercise in pregnancy: First trimester risks. Clin. Obstet. Gynecol..

[36] Giannini DT, Kuschnir MCC, de Oliveira CL, Szklo M (2017). Waist-to-height ratio as a predictor of C-reactive protein levels. J. Am. Coll. Nutr..

[37] Grimstad F, Krieg S (2016). Immunogenetic contributions to recurrent pregnancy loss. J. Assist. Reprod. Genet..

[38] Sindhu S (2015). Obesity is a positive modulator of IL-6R and IL-6 expression in the subcutaneous adipose tissue: Significance for metabolic inflammation. PLoS ONE.

[39] Triunfo S, Lanzone A (2014). Impact of overweight and obesity on obstetric outcomes. J. Endocrinol. Investig..

[40] Maconochie N, Doyle P, Prior S, Simmons R (2007). Risk factors for first trimester miscarriage—results from a UK-population-based case-control study. BJOG.

[41] Pineles BL, Park E, Samet JM (2014). Systematic review and meta-analysis of miscarriage and maternal exposure to tobacco smoke during pregnancy. Am. J. Epidemiol..

[42] Lambers DS, Clark KE (1996). The maternal and fetal physiologic effects of nicotine. Semin. Perinatol..

[43] Andersen AM, Andersen PK, Olsen J, Gronbaek M, Strandberg-Larsen K (2012). Moderate alcohol intake during pregnancy and risk of fetal death. Int. J. Epidemiol..

[44] Sundermann AC (2019). Alcohol use in pregnancy and miscarriage: A systematic review and meta-analysis. Alcohol Clin. Exp. Res..

[45] Mamluk L (2020). Evidence of detrimental effects of prenatal alcohol exposure on offspring birthweight and neurodevelopment from a systematic review of quasi-experimental studies. Int. J. Epidemiol..

[46] NHS. Drinking alcohol while pregnant. https://www.nhs.uk/conditions/pregnancy-and-baby/alcohol-medicines-drugs-pregnant/ (2020).

[47] Bech BH, Nohr EA, Vaeth M, Henriksen TB, Olsen J (2005). Coffee and fetal death: A cohort study with prospective data. Am. J. Epidemiol..

[48] Greenwood DC (2010). Caffeine intake during pregnancy, late miscarriage and stillbirth. Eur. J. Epidemiol..

[49] Tolstrup JS (2003). Does caffeine and alcohol intake before pregnancy predict the occurrence of spontaneous abortion?. Hum. Reprod..

