# Supplementary material for: Systematic review and meta-analysis of female lifestyle factors and risk of recurrent pregnancy loss
Source: Sci Rep. 2021 Mar 29;11:7081. doi: 10.1038/s41598-021-86445-2 (PMC8007745; doi:10.1038/s41598-021-86445-2)
Supplement: Supplementary file 1 — Supplementary Information. [file 41598_2021_86445_MOESM1_ESM.pdf]

Title: Systematic review and meta-analysis of female lifestyle factors and risk of recurrent pregnancy loss

Authors: Ka Ying Bonnie Ng[1,2], George Cherian[2], Alexandra J. Kermack[1,2], Sarah Bailey[2], Nick Macklon[3,4], Sesh K Sunkara[5], Ying Cheong*[1,2,6]

[1]School of Human development and Health, Faculty of Medicine, University of Southampton, Southampton SO16 6YD, UK

[2]Department of Obstetrics and Gynaecology, Princess Anne Hospital, Room F86, Level F, Coxford Road, Southampton, SO16 5YA, UK

[3]Zealand University Hospital, University of Copenhagen, Denmark

[4]London Women's Clinic, London, UK, W1G 6AP

[5]Department of Women and Children's Health, Kings College London, 11th Floor, Tower Wing, Guy's Hospital, SE1 9RT, UK

[6]Complete Fertility Southampton, Princess Anne Hospital, Coxford Road, SO16 5YA

Corresponding author:

Professor Ying Cheong

School of Human development and Health, Faculty of Medicine, University of Southampton, Southampton SO16 6YD, UK

Department of Obstetrics and Gynaecology, Princess Anne Hospital, Room F86, Level F, Coxford Road, Southampton, SO16 5YA, UK

Complete Fertility Southampton, Princess Anne Hospital, Coxford Road, SO16 5YA

Email: y.cheong@soton.ac.uk
Telephone: 023 8120 6033

Supplementary Method - Search Strategy

1. exp Abortion, Spontaneous/
2. (recurrent adj3 miscarr$).mp.
3. miscarr$.mp.
4. 1 or 2 or 3
5. exp Life Style/
6. exp Diet/
7. nutrition disorders/ or exp malnutrition/ or exp overnutrition/
8. Caffeine/
9. Smoking/
10. Marijuana Smoking/
11. exp Alcohol Drinking/
12. exp Street Drugs/
13. exp Exercise/
14. exp "Body Weights and Measures"/
15. Stress, Psychological/
16. Work Schedule Tolerance/
17. Lifting/
18. diet$.mp.
19. (food adj3 intake).mp.
20. Folic Acid/
21. folate.mp.
22. folic.mp.
23. exp Vitamins/
24. vitamin$.mp.
25. caffeine.mp.
26. smoking.mp.
27. alcohol.mp.
28. recreational drugs.mp.
29. cocaine.mp.
30. marijuana.mp.

31. exercise.mp.
32. (physical adj3 activit$).mp.
33. run$.mp.
34. swim$.mp.
35. yoga.mp.
36. pilates.mp.
37. jog.mp.
38. jogging.mp.
39. cycling.mp.
40. cycle.mp.
41. riding.mp.
42. hiking.mp.
43. climb$.mp.
44. sail$.mp.
45. skiing.mp.
46. underweight.mp.
47. overweight.mp.
48. BMI.mp.
49. body mass.mp.
50. under weight.mp.
51. over weight.mp.
52. stress$.mp.
53. lifting.mp.
54. shift work.mp.
55. 5 or 6 or 7 or 8 or 9 or 10 or 11 or 12 or 13 or 14 or 15 or 16 or 17 or 18 or 19 or 20 or 21 or 22 or 23 or 24 or 25 or 26 or 27 or 28 or 29 or 30 or 31 or 32 or 33 or 34 or 35 or 36 or 37 or 38 or 39 or 40 or 41 or 42 or 43 or 44 or 45 or 46 or 47 or 48 or 49 or 50 or 51 or 52 or 53 or 54
56. 4 and 55

Supplementary Table S1

Author and Year	Study type	Population	Definition of Recurrent pregnancy loss (RPL)	Population numbers	Primary Outcome	Other/Secondary Outcomes
Bhandari 2016 [12]	Retrospective cohort	Women with history of RPL who attended tertiary implantation clinic in Warwick, UK	≥3 consecutive miscarriages <24 weeks gestation	414 cases	Difference in length of time to pregnancy interval in obese and normal BMI women with RPL	Relationship between pregnancy loss patterns and BMI
Boots 2014 [13]	Prospective cohort	Women with RPL who attended University of Chicago, USA	≥2 miscarriages <10 weeks gestation (not stated whether consecutive)	372 cases	Association between obesity and further euploid miscarriage in women with RPL	Association between obesity and further male euploid miscarriage in women with RPL
George 2006 [14]	Population-based case-control study	Women who attended O&G department in Uppsala Hospital, Sweden	≥2 successive miscarriages <12 weeks gestation	691 (108 cases, 583 controls)	Identification of various risk factors for RPL including BMI, smoking, alcohol, caffeine, folate supplementation and shift work	-
Jung 2015 [15]	Retrospective cohort	Women who had taken part in the large-scale genomic cohort study (HEXA) in Korea	≥2 miscarriages <20 weeks gestation (not stated whether consecutive)	80,446 (1704 cases, 78742 controls)	Effect of BMI at age 18-20 on risk of spontaneous abortion and RPL later in life	Effect of BMI at age 18-20 on age of first miscarriage. Relationship between various factors and risk of spontaneous abortion including education and smoking

Kolte 2015 [16]	Cross sectional study	Women referred to the Danish RPL Unit at the Fertility Clinic at Rigshospitalet, Denmark	≥3 consecutive miscarriages <12 weeks gestation	2114 (301 cases, 1813 controls)	Prevalence of stress and depression measured via MDI and PSS	-
Lashen 2004 [17]	Nested case-control	Obese and normal BMI women who attended Solihull Maternity Unit, UK	>3 successive miscarriages <12 weeks gestation	4932 (1644 cases, 3288 controls)	Association of obesity with risk of first trimester and recurrent miscarriage	Prevalence of gestational diabetes in obese and normal weight women
Li 2012 [18]	Prospective case-control	Women who attended O&G department in Royal Hallamshire Hospital, Sheffield, UK	≥3 unexplained consecutive miscarriages (gestation not stated)	85 (45 cases, 40 controls)	Association between stress and RPL	Impact of stress on subsequent pregnancy in women with RPL. Association between stress and its biochemical markers
Lo 2012 [19]	Prospective cohort	Women with RPL referred to miscarriage clinic in St Mary's Hospital, UK	≥3 unexplained consecutive miscarriages (gestation not stated)	696 cases	Association between BMI and future pregnancy outcome in women with RPL	Risk of further miscarriage in Asian women with RPL
Matjila 2017 [20]	Retrospective cohort	Women attending RPL clinic at the Groote Schuur	≥3 consecutive first trimester miscarriages	592 cases	Prevalence of commonly investigated medical conditions (e.g. PCOS, IGT,	Association between BMI and RPL

		Hospital's, Cape Town, South Africa			Type 2 Diabetes) associated with RPL	
Metwally 2010 [21]	Prospective cohort	Women with history of RPL referred to miscarriage clinic at Sheffield Teaching Hospitals, UK	≥3 successive miscarriages <20 weeks gestation	844 pregnancies from 491 women	Effect of BMI on risk of further miscarriage in women with RPL	Effect of other factors on further miscarriage including maternal age, PCOS and number of previous miscarriages
Parazzini 1991 [22]	Retrospective case-control	Women referred to O&G clinic in Milan, Italy	≥2 successive unexplained miscarriages <3 months gestation	270 (94 cases, 176 controls)	Risk factors for RPL including smoking, caffeine intake and alcohol consumption	-
Parazzini 1990 [23]	Retrospective case-control	Women referred to O&G clinic in Milan, Italy	≥2 successive unexplained miscarriages <3 months gestation	270 (94 cases, 176 controls)	Relative risk of RPL with alcohol consumption	Relative risk of RPL with various factors including caffeine intake and smoking
Peppone 2009 [24]	Survey	Caucasian women who had attended Roswell Park Cancer Institute, USA	≥2 foetal losses. Foetal loss = miscarriage or stillbirth. (whether successive not stated)	4804 (596 cases, 4208 controls)	Association between second hand smoke exposure with various pregnancy difficulties including foetal loss and difficulty becoming pregnant	-
Stefanidou 2011 [25]	Retrospective case-control	Caucasian women who attended O&G	≥3 unexplained consecutive miscarriages	312 (52 cases, 260 controls)	Association between risk of RPL and caffeine consumption	Association between risk of RPL and BMI, smoking,

		department in University of Turin, Italy	<20 weeks gestation			alcohol and environmental exposure
Ticconi 2010 [26]	Retrospective case-control	Women who attended University Hospitals in Rome, Italy	≥3 miscarriages (gestation or whether consecutive not stated)	219 (133 cases, 86 controls)	Association between BMI and RPL	-
Zhang 2010 [27]	Retrospective case-control	Women who attended O&G institute and pregnancy clinics in Guangzhou, China	≥3 unexplained miscarriages <20 weeks gestation. (whether successive not stated)	726 (326 cases, 400 controls)	Identification of various risk factors for RPL including BMI, smoking, alcohol and caffeine	-

Supplementary Table S1 Main summary of all studies included in this review (17 studies)
BMI = body mass index, IGT = Impaired Glucose Tolerance, O&G = Obstetrics and Gynaecology, PCOS = polycystic ovarian syndrome, RPL = recurrent pregnancy loss, MDI = Major Depression Index, PSS = Cohen's Perceived Stress Scale

Supplementary Table S2
a)

Author and Year	Groups (BMI, kg/m^2)	Findings
Bhandari 2016 [12]	18.5-24.9<br>25-29.9<br>≥30	Higher pregnancy rates for obese women with RPL. 65.2% and 80% within 3 and 6 months, respectively, compared to 50% and 70% for overweight women and 49.2% and 65.8% for normal BMI
Boots 2014 [13]	<30 (ref, 1.0)<br>≥30	Significantly higher rate of further euploid miscarriage in obese women (RR 1.63, 95% CI 1.08-2.47; p=0.02)
George 2006* [14]	<20 (ref, 1.0)<br>20 – 24.9<br>25 – 29.9<br>≥30	No significant difference in risk of RPL between groups (OR 1.2, 95% CI 0.5-2.6 for overweight group and 1.5, 95% CI 0.6-3.6 for obese group)
Jung 2015* [15]	18.5-22.9 (ref, 1.0)<br>23-24.9<br>≥25	RPL significantly more common if obese at age 18-20 (OR 1.39, 95% CI 1.09-1.78)<br>Obesity increased risk of miscarriage at ≤25 years age (OR 1.22, 95% CI 1.02-1.45)
Lashen 2004 [17]	19 - 24.9 (ref, 1.0)<br>>30	Obese group had significantly higher risk of RPL (OR 3.51, 95% CI 1.03 – 12.01; p=0.04) and early miscarriage (OR 1.2, 95% CI 1.01-1.46; p=0.04)
Lo 2012* [19]	18.5-24.9 (ref, 1.0)<br>25-29.9<br>≥30	Obesity significantly increases risk of further miscarriage (OR 1.73, 95% CI 1.06-2.83; p=0.028). No significant difference if overweight (OR 1.27, 0.89-1.83)<br>Asian women had higher risk of further miscarriage (OR 2.87, 95% CI 1.52-5.39)
Matjila 2017 [20]	<18.5<br>18.5-24.9<br>25-29.9<br>≥30	The mean BMI in the RPL population was 29.58 ± 6.96 (mean ± SD), with 95% CI (29.01, 30.15). 73% were overweight and obese, and 42% had class I, II and III obesity.
Metwally 2010* [21]	19-24.9 (ref, 1.0)<br>25-29.9<br>30-34.9<br>≥35	Risk of further miscarriage significantly higher if obese (OR 1.71, 95% CI 1.05-2.8). No significant difference if overweight (OR 1.02, 95% CI 0.72-1.45).<br>Miscarriage rate in unexplained recurrent miscarriage subgroup highest in obese patients with 60%, compared to 40% in women with normal BMI
Stefanidou 2011 [25]	18.5-25<br>25-30<br>≥30	BMI not significantly different between cases and controls
Zhang 2010 [27]	≤23.9 (ref, 1.0)	Women with RPL more likely to have BMI ≥24 (OR 1.55, 95% CI 1.12-2.14)

	≥24	

b)

**Author and Year**	**Groups (BMI, kg/m²)**	**Findings**
George 2006* [14]	<20 (ref, 1.0)<br>20 – 24.9<br>25 – 29.9<br>≥30	No significant difference in risk of RPL between groups
Jung 2015* [15]	<18.5<br>18.5-22.9 (ref, 1.0)	RPL significantly more common if underweight at age 18-20 (OR 1.29, 95% CI 1.14-1.46)<br>Being underweight resulted in miscarriage at ≥26 years age (OR 1.17, 95% CI 1.08-1.27)
Lo 2012* [19]	<18.5<br>18.5-24.9 (ref, 1.0)	No significant difference in further risk of miscarriage if underweight (OR 0.12, 95% CI 0.15-1.0)
Metwally 2010* [21]	<19<br>19-24.9 (ref, 1.0)	Risk of further miscarriage significantly higher if underweight (OR 3.98, 95% CI 1.06-14.92)
Parazzini 1991* [22]	<20 (ref, 1.0)<br>20-22.5<br>>22.5	No significant relationship between BMI and risk of RPL (RR 1.3, 95% CI 0.7-2.5 for BMI 20-22.5 and 1.1, 95% CI 0.6-2.0 for BMI >22.5)
Ticconi 2010 [26]	Women without RPL<br>Women with RPL	Women with RPL had significantly lower BMI (23.02 +/- 3.87 vs. 26.39 +/- 5.92, p<0.001)

Supplementary Table S2 Recurrent pregnancy loss and association with being a) BMI>25/BMI>30 (10 studies) and b) being underweight (6 studies)

Included in meta-analyses*

Ref = reference group; OR = odds ration; CI = confidence interval; RPL = recurrent pregnancy loss

Supplementary Table S3
a)

**Author and Year**	**Groups**	**Findings**
George 2006* [14]	Non-smoker<br>Smoker	Smoking significantly increased risk of RPL (OR 2.1, 95% CI 1.1-4.1; p = 0.02)
Parazzini 1991* [22]	Never smokers (ref, 1.0)<br>Current smokers (cigarettes /day)<br>- 1-9<br>- ≥10<br><br>Ex-smokers	Increased risk of RPL if current smoker but not significant (RR 1.4, 95% CI 0.8-2.9)<br>Risk of RPL increased with number of cigarettes smoked and this *trend* significant (p=0.04)
Peppone 2009 [24]	No SHS (ref, 1.0)<br>Childhood SHS but no adult SHS<br>Adult SHS but no Childhood SHS<br>Both Adult and Childhood SHS	Significant association between RPL and SHS during both childhood and as adult (OR 1.62, 95% CI 1.25-2.11)
	Adult SHS hours/day:<br>- None (ref, 1.0)<br>- 0.5-1.5<br>- 2-5<br>- ≥6	Significant positive trend between increasing SHS hours and odds of RPL (P<0.05)
	Age of starting to smoke:<br>- Never smokers (ref, 1.0)<br>- 18-23<br>- 16-17<br>- 15<br>- 14<br>- 13<br>- ≤12	Risk of RPL significantly increased with younger age of starting to smoke (OR 1.7, 95% CI 1.1-2.6 for 18-23 y/o; OR 1.8, 95% CI 1.2-2.7for 14 y/o; OR 2.3, 95% CI 1.6-3.5 for ≤12 y/o)
Stefanidou 2011 [25]	Non-smoker<br>Smoker	Significant association between RPL and smoking (OR 2.857, 95% CI 1.25-6.532; p=0.018)

Zhang 2010* [27]	Active smoking (cigarettes/day):<br>- Never (ref, 1.0)<br>- ≤9<br>- 10-19<br>- ≥20	Non-significant increase in risk of RPL with more cigarettes smoked (OR 1.42, 95% CI 0.12-17.17 for ≤9 cigarettes, OR 1.62, 95% CI 0.13-20.87 for 10-19 cigarettes, OR 2.11, 95% CI 0.09-47.66 for ≥20 cigarettes)
	Passive smoking (hours/day):<br>- Never (ref, 1.0)<br>- <1<br>- ≥1	Significant increase in risk of RPL with more passive smoke exposure (OR 2.30, 95% CI 1.50-3.52 with <1 hour, OR 4.75, 95% CI 3.23-6.99 with ≥1 hour). Trend significant, p<0.001

b)

**Author and Year**	**Groups**	**Findings**
George 2006* [14]	Non-drinker<br>≥0.1cL/week	No significant increase in risk of RPL with alcohol (OR 1.3, 95% CI 0.7-2.7)
Parazzini 1990* [23]	Maternal:<br>Non-drinkers (ref, 1.0)<br>Average <2 drinks/day<br>Average ≥2 drinks/day<br>(1 drink = 12g pure alcohol)	Alcohol consumption not associated with risk of RPL(RR 0.9, 95% CI 0.6-1.5 for <2 drinks/day and RR 0.8, 95% CI 0.4-1.6 for ≥2 drinks/day)
	Paternal:<br>Non-drinkers (ref, 1.0)<br>Average <3 drinks/day<br>Average ≥3 drinks/day<br>(1 drink = 12g pure alcohol)	Non-significant increase in risk of RPL with alcohol (RR 1.7, 95% CI 0.7-4.0 for <3 drinks/day and RR 1.4, 95% CI 0.7-2.8 for ≥3 drinks day)
Stefanidou 2011 [25]	Non-drinker<br>Drinker	Women with RPL consumed less alcohol than controls (OR 0.289, 95% CI 0.11-0.756; p=0.007)
Zhang 2010* [27]	Non-drinkers (ref, 1.0)<br><5 units/week<br>≥5 units/week	No significant difference in risk of RPL between groups (OR 0.83, 95% CI 0.46-1.49 with <5 units and OR 0.84, 95% CI 0.43-1.64 with ≥5 units)

c)

**Author and Year**	**Groups**	**Findings**
George 2006* [14]	Caffeine intake (mg/day) during pregnancy:<br>- 0-99 (ref, 1.0)<br>- 100-299<br>- ≥300	Non-significant increase in risk of RPL with increased caffeine intake (OR 1.8, 95% CI 0.8-3.9 for ≥300mg/day).<br>Significant increase in risk of RPL with increased caffeine intake in non-smokers (OR 2.7, 95% CI 1.1-6.2 for ≥300mg/day) but not smokers (OR 0.4, 95% CI 0.05-4.1 for ≥300mg/day)
Parazzini 1990* [23]	Coffee drinkers<br>Coffee non-drinkers	No difference in risk of RPL with caffeine intake (RR 1.4, 95% CI 0.7-2.6)
Stefanidou 2011 [25]	Caffeine intake (mg/day)<br>- ≤150.9 (ref, 1.0)<br>- 151-300.9<br>- ≥301	Significantly increased risk of RPL with increased caffeine intake (OR 3.045, 95% CI 1.237-7.287 for 151-300.9mg/day; p=0.012 and OR 16.106, 95% CI 6.547-39.619 for ≥301; p=<0.00)
Zhang 2010* [27]	Coffee non-drinkers (ref, 1.0)<br>Coffee drinkers (mg/day):<br>- ≤99<br>- 100-299<br>- ≥300	No significant difference in risk of RPL between groups (OR 2.55, 95% CI 0.44-14.74 for ≤99 mg/day, OR 2.39, 95% CI 0.39-14.80 for 100-299mg/day and OR 2.76, 95% CI 0.45-16.95 for ≥300mg/day)

Supplementary Table S3 Recurrent pregnancy loss and association with a) smoking (6 studies), b) alcohol intake (5 studies) and caffeine intake (4 studies)

Included in meta-analyses*

Ref = reference group; RR = relative risk; OR = odds ration; CI = confidence interval; RPL = recurrent pregnancy loss; SHS = second hand smoke exposure

Supplementary Table S4
a)

Author and Year	Groups	Findings
Kolte 2015 [16]	Moderate/severe depression and<br>High stress (score ≥19 on PSS) in RPL and controls	8.6% of RPL patients (compared to 2.2% in the controls) had MDI scale corresponding to an ICD-10 moderate or severe depression, OR 5.53 (CI 2.09, 14.61) adjusted for age, education, household income, number of live born children and prior pregnancies.<br>41.2% of RPL patients reported a high stress level (compared to 23.2% in the controls), adjusted OR 1.59 (95% CI 1.03, 2.44)<br>Mean scores on PSS: 16.71 in RPL, 13.97 in controls, mean difference 2.74 (95% CI 1.94, 3.53).
Li 2012 [18]	Psychological stress in fertile women<br>Psychological stress in women with RPL	Women with RPL had significantly higher scores of psychological stress:<br>FPI OR 1.02, 95% CI 1.01-1.04, $p<0.05$<br>PSS OR 1.13, 95% CI 1.13-1.24, $p<0.05$<br>PPA OR 0.89, CI 0.82-0.96, $p<0.05$<br>PNA OR 1.12, 95% CI 1.04-1.21, $p<0.05$
	Biochemical stress markers in fertile women<br>Biochemical stress markers in women with RPL	No significant increase in RPL group:<br>Peripheral CD^{dim}NK cells OR 1.01, 95% CI 0.97-1.05<br>Peripheral CD^{bright}NK cells OR 1.14, 95% CI 0.65-1.98
	Psychological stress in women with RPL with<br>further miscarriage<br><br>Psychological stress in women with RPL with subsequent live birth	Moderate stress (lower PPA score) associated with better pregnancy outcome (OR 1.17, 95% CI 1.03-1.33; $p<0.05$)

b)

**Author and Year**	**Groups**	**Findings**
George 2006 [14]	No folate supplementation<br>Folate supplementation (>400mcg/day)	Significantly increased risk of RPL in folate supplementation group (OR 3.1, 95% CI 1.4-6.6, p=0.004). Women taking supplements older (p=0.02) and took longer to conceive (p<0.01)
	Plasma folate (nmol/L):<br>- ≤4.9<br>- 5.0-8.9 (ref, 1.0)<br>- 9.0-13.9<br>- ≥14	No significant dose-dependent association between plasma folate and RPL:<br>≤4.9 nmol/L OR 0.8, 95% CI 0.4-1.9<br>9-13.9 nmol/L OR 2.3, 95% CI 1.1-4.6<br>≥14 nmol/L OR 2.2, 95% CI 1.0-4.9

c)

**Author and Year**	**Groups**	**Findings**
George 2006 [14]	No shift work<br>Shift work	No significant increase in RPL with shift work (OR 1.3, 95% CI 0.5-3.0)

Supplementary Table S4 Recurrent pregnancy loss and association with a) stress (2 studies), b) nutritional supplementation (1 study) and c) shift work (1 study)

OR = odds ratio; CI = confidence interval; RPL = recurrent pregnancy loss; FPI = fertility problem inventory; PSS = perceived stress scale; PPA = positive and negative affect scale positive affect; PNA = positive and negative affect scale negative affect; NK = natural killer cells, MDI = Major Depression Index, PSS = Cohen's Perceived Stress Scale, ICD-10 = 10th revision of the International Statistical Classification of Diseases and Related Health Problems
